# A Case of Paraneoplastic Dermatomyositis Presenting With Malignant Pleural Effusion

**DOI:** 10.7759/cureus.39070

**Published:** 2023-05-16

**Authors:** Puja Upadhyay, Ulhas Jadhav, Pankaj Wagh, Babaji Ghewade, Anjana Ledwani

**Affiliations:** 1 Respiratory Medicine, Jawaharlal Nehru Medical College, Datta Meghe Institute of Higher Education and Research, Wardha, IND

**Keywords:** lung, malignancy, adenocarcinoma, pleural effusion, dermatomyositis

## Abstract

Dermatomyositis is an uncommon inflammatory condition characterized by proximal muscle weakness with distinct cutaneous manifestations. Like any other systemic disease, it affects multiple organs, the lungs being one of them. Common pulmonary manifestations of dermatomyositis (DM) include interstitial lung disease (ILD), primary lung malignancy, and aspiration pneumonia. The involvement of the pleura is not commonly seen, and pleural effusion is rarely reported in DM. Its presence should prompt further workup, especially for malignancy. An association between dermatomyositis and malignancy has been studied widely and is well established. Here, we report a 37-year-old female with classical cutaneous manifestations and myopathy of dermatomyositis presenting with a malignant left-sided pleural effusion.

## Introduction

Dermatomyositis (DM) is one of several types of idiopathic inflammatory myopathy with distinct involvement of the skin [1]. It is thought to affect 9.63 out of every one million individuals. Women are at twice as much risk as men [2]. The diagnosis is made using clinical signs like a typical skin rash, progressive muscle weakness, a raised level of the muscle enzyme in serum, the presence of myositis-specific antibodies in the blood, aberrant electromyograms, and results from muscle and/or skin biopsies [1].

Individuals with classic dermatomyositis typically show symmetrical proximal muscle weakening and characteristic cutaneous lesions. V-sign, shawl sign, Gottron's papules, and heliotrope rash are common dermal manifestations of the disease. In addition, periungual telangiectasias, palmar papules on the joint creases, cuticular overgrowth, calcinosis, and poikiloderma are frequently seen in DM patients. Pruritus and sensitivity to sunlight are other common cutaneous symptoms [3].

Pulmonary involvement in dermatomyositis includes interstitial lung disease, bronchiolitis obliterans, organizing pneumonia, bronchopneumonia, pulmonary vasculitis, pulmonary edema, primary lung malignancy, diffuse alveolar damage, pulmonary embolism, and diaphragmatic atrophy. An accumulation of significant pleural effusion is rare in individuals with DM and should prompt further evaluation, especially for malignancy [4].

The associated risk of malignancy with DM and its presentation as a paraneoplastic syndrome have been well documented. Most of the reported cases of DM are idiopathic. Still, around fifteen to thirty percent are a manifestation of paraneoplastic syndromes of an underlying malignancy, which can include cancer of the stomach, lungs, ovaries, pancreas, stomach, non-Hodgkin lymphoma, and colorectal cancer [5].

The case going to be described here is of a 37-year-old female with classical cutaneous manifestations and myopathy of dermatomyositis presenting to us with a massive left-sided pleural effusion, which upon further evaluation was found to be a malignant effusion secondary to adenocarcinoma of the left lung.

## Case presentation

A 37-year-old woman came to us with the primary symptom of breathlessness for three months, which was initially modified Medical Research Council (mMRC) grade I and had increased to grade II-III in the last 15 days. Upon further inspection, the patient had dark purple hyperpigmented rashes over her face, which were also present over her chest, knuckles, elbow, knees, and back. On further inquiry, the patient revealed to have had rashes on and off for the last six months, which were painful, associated with itching and worsened on exposure to sunlight. She also had a history of painful joint swelling for six months, facial swelling for six months, and difficulty getting up from a sitting position for one month. She further gave a history of low-grade fever for one month and myalgia for one month. The patient visited several local hospitals over the last six months for the above-mentioned complaints, where she was managed conservatively and no specific diagnosis was made.

**Figure 1:**
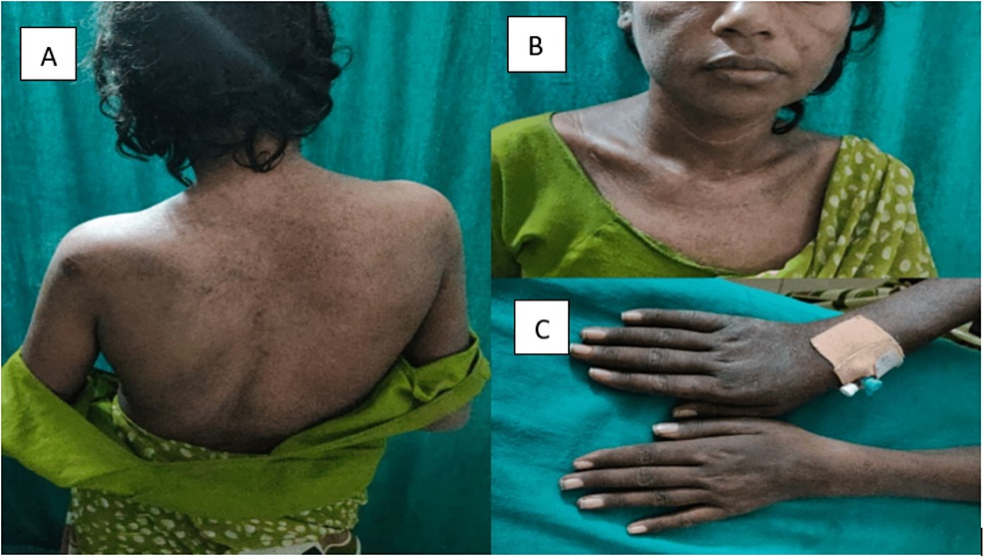
Hyperpigmented dark purple rashes across the cheek (B), neck (B), back (A), and knuckles (C)

On general examination, the patient was moderately built, with a BMI of 22 kilograms per square meter. She had a pulse rate of 80 beats per minute, blood pressure of 130/90 millimeters of mercury in her right arm in the supine position, a respiratory rate of 24 cycles per minute, and oxygen saturation of 92 percent in room air, needing two liters of oxygen support. On auscultation, she had absent breath sounds in the left mammary, inframammary, axillary, infraaxillary, scapular, and interscapular areas. The patient had dark purple hyperpigmented rashes on her eyelids, cheeks, across the nose bridge, chest, back, knuckles, and knees. The patient also had roughening, and cracking of the skin over the sides and tips of the digits, resembling that of a manual laborer's or mechanic’s hand. These cutaneous manifestations are shown in Figure 1.

**Figure 2:**
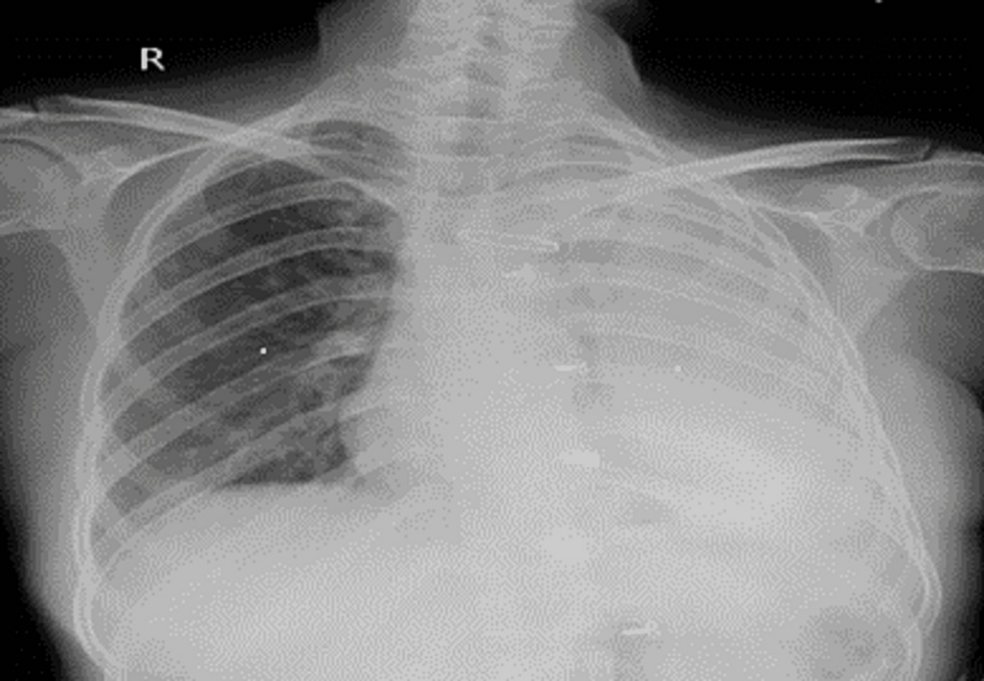
Chest X-ray showing left opaque hemithorax

Neurological examination revealed bilateral lower limb proximal muscle weakness. History and general examination raised suspicion of dermatomyositis with pleural effusion. Further investigations were done to confirm the diagnosis. Her blood investigation revealed an elevated creatine kinase level of 900 units/L. The rest of her blood investigations were unremarkable, including the antinuclear antibody (ANA), which was negative. An erect X-ray of the chest was obtained, which revealed a left opaque hemithorax with a shift of the mediastinum to the right, as shown in Figure 2, suggesting massive left pleural effusion. It was confirmed with ultrasonography of the thorax.

**Figure 3:**
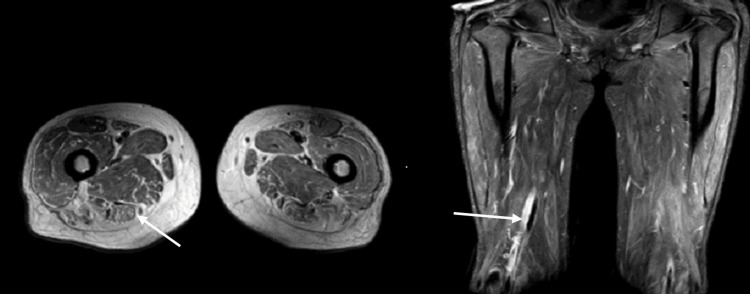
MRI of the thigh showed high T2/STIR signals throughout the musculature of the thigh and a visualized pelvis suggestive of myositis. (A) Transverse plane; (B) Coronal plane MRI: magnetic resonance imaging; STIR: short inversion time inversion recovery

MRI of the thigh showed findings suggestive of myositis in both thighs, as shown in Figure 3.

Electromyogram (EMG) was done in the left tibialis anterior, right bicep, and right first dorsal interosseous (FDI), suggesting a myopathic pattern. The result is summarized in Table 1.

**Table 1:** Summary of EMG report EMG: electromyography; MUP: motor unit potential; FDI: first dorsal interosseus

EMG REPORT	
Left tibialis anterior	No spontaneous activity, with myopathic Interference pattern and low voltage MUP.
Left FDI	No spontaneous activity, with normal interference pattern and low voltage MUP.
Left Bicep	No spontaneous activity, with normal interference pattern and low voltage MUP.

**Figure 4:**
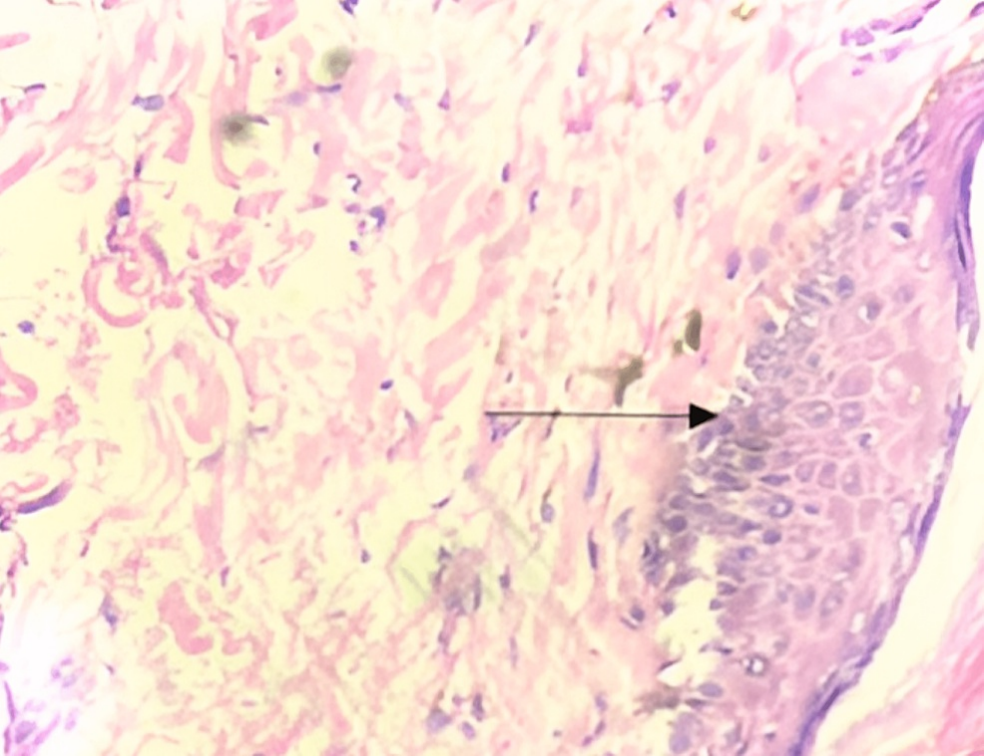
HPE of skin showing basal vacuolization (marked with arrow) HPE: histopathological examination

A skin biopsy was done and sent for histopathological examination (HPE), which showed an atrophied epidermis with minimal keratosis, loss of rete ridges, and dermal papillae. The basal layer showed basal vacuolation consistent with dermatomyositis, as shown in Figure 4. The reticular dermis showed dermal edema with reduced adnexal structure.

Therapeutic thoracocentesis was done, and pleural fluid was sent for routine investigation, which showed a high lactate dehydrogenase (LDH) level. The results are summarized in Table 2.

**Table 2:** Pleural fluid analysis report. TLC: total leukocyte count; cells/cumm: cells per cubic millimeter; DLC: differential leukocyte count; U/L: unit per liter; g/L: gram per liter; LDH: lactate dehydrogenase; ADA: adenosine deaminase

PLEURAL FLUID PARAMETERS(UNIT)	RESULT	NORMAL RANGE
TLC (cells/cumm)	Approximately 316	< 1000
DLC	Polymorphs= 8% Lymphocytes= 20%	Polymorphs = 2 - 5% Lymphocyte = 5 - 10%
PH	7.4	7.60 – 7.64
LDH (U/L)	9108	100 - 200
Protein (g/L)	7	10 - 20
ADA (U/L)	14	> 40
Trunat	Negative	Negative
Culture	No Growth	No Growth

**Figure 5:**
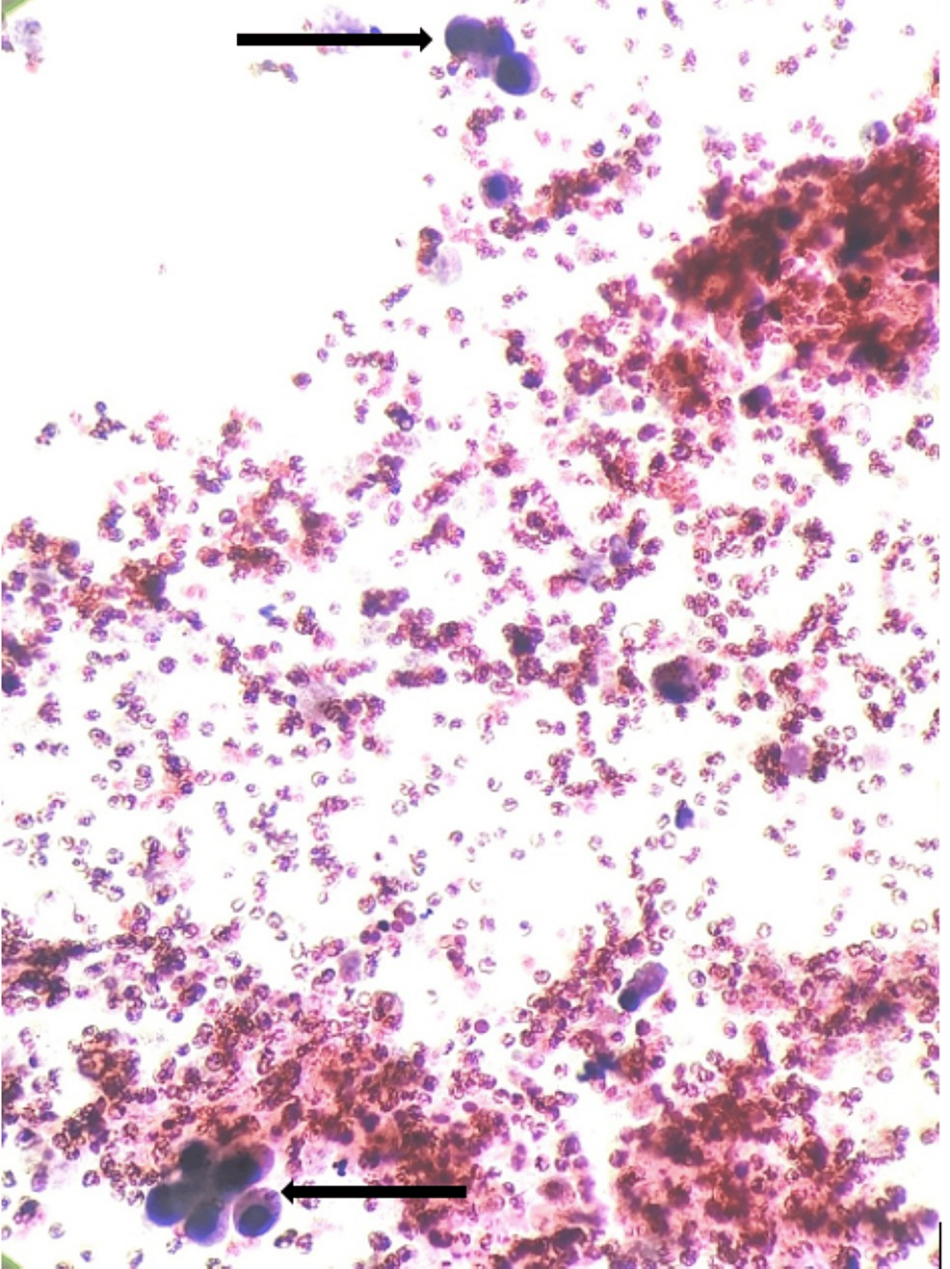
Pleural fluid cytology showing infiltrates of adenocarcinoma (marked with black arrow)

Pleural fluid cytology suggested "infiltrates of adenocarcinoma," as shown in Figure 5, which was confirmed with a pleural biopsy. The histopathological examination of the pleura showed fibrocollagenous tissue with focal deposits of malignant epithelial cells. The immunohistochemical study was positive for thyroid transcription factor 1 (TTF-1) and napsin A, confirming the diagnosis of primary lung adenocarcinoma..

A diagnosis of malignant pleural effusion secondary to adenocarcinoma of the lung with dermatomyositis as a paraneoplastic syndrome was made. The patient was started on glucocorticoids for dermatomyositis and was referred to an oncologist for chemotherapy. She was advised to undergo four cycles of chemotherapy with carboplatin and pemetrexed three times weekly and then re-evaluate for a response.

## Discussion

Dermatomyositis falls under a spectrum of diseases collectively called idiopathic inflammatory myopathy [6]. This collection of autoimmune diseases is characterized by chronic muscle inflammation and weakness [2]. Classical cutaneous manifestations in DM include a red-purple rash on the eyelids, face, back, neck, knuckles, and knee. Some typical cutaneous signs are Gottron's papules, heliotrope rash, the V-neck sign, the shawl sign, cuticular overgrowth, and photosensitivity [7]. DM causes classical symmetrical proximal muscle weakness. The patient complains of difficulty getting up from chairs, ascending stairs, and/or shaving or combing one’s hair.

Dermatomyositis increases the chance of cancer sixfold compared to the general population. DM with concurrent malignancy accounts for 6% to 60% of all cases [8]. Breast, colon, lung, ovarian, melanoma, non-Hodgkin lymphoma (NHL), nasopharyngeal, and stomach are the most commonly associated malignancies in patients with DM [9]. Non-small cell carcinoma (adenocarcinoma) of the lung, ovary, or gastrointestinal tract is more prevalent in countries from the West, and nasopharyngeal carcinoma is found more in Southern China, Southeast Asia, and Northern Africa. The most common type of DM-related lung cancer is small-cell lung cancer (29%), followed by squamous cell carcinoma (21%), and then adenocarcinoma (8%) [9]. The reaction of the immune system to antigens expressed in both cancer cells and regenerating fibers in affected muscle can be hypothesized as a reason for the paraneoplastic phenomenon [10].

This case report is of a female with the typical cutaneous and myopathic manifestations of dermatomyositis who presented with the chief complaint of dyspnea owing to massive left-sided pleural effusion. The diagnosis of DM in our case was made based on classical cutaneous manifestations such as Gottron's papules, proximal muscle weakness with electromyography (EMG) suggestive of a myopathic pattern, a skin biopsy suggesting vacuolar degeneration of the basal layer of the epidermis, an MRI thigh finding suggestive of myositis, and elevated muscle enzyme levels. The patient developed these symptoms around the same time as her dyspnea, which was due to malignant pleural effusion, suggesting that the dermatomyositis in this particular case was a paraneoplastic syndrome secondary to an underlying lung malignancy.

## Conclusions

Dermatomyositis is an uncommon condition, but its association with malignancy both as a premalignant condition and as a paraneoplastic syndrome is widely established. A high suspicion index for malignancy should be maintained when dealing with a case of dermatomyositis, especially with uncommon manifestations like pleural effusion, as highlighted in our case.
